# Diabetesschulung und -beratung bei Erwachsenen mit Diabetes (Update 2023)

**DOI:** 10.1007/s00508-022-02120-0

**Published:** 2023-04-20

**Authors:** Raimund Weitgasser, Christian Ciardi, Julia Traub, Merlena Barta, Michaela Riedl, Martin Clodi, Bernhard Ludvik

**Affiliations:** 1 Kompetenzzentrum Diabetes, Privatklinik Wehrle-Diakonissen, Salzburg, Österreich; 2 Abteilung für Innere Medizin, Krankenhaus St. Vinzenz, Zams, Österreich; 3 Ernährungsmedizinischer Dienst, LKH Univ. Klinik Graz, Graz, Österreich; 4 1. Medizinische Abteilung mit Diabetologie, Endokrinologie und Nephrologie, Klinik Landstraße, Wien, Österreich; 5 Universitätsklinik für Innere Medizin III, Medizinische Universität Wien, Wien, Österreich; 6 Abteilung für Innere Abteilung, Konventhospital der Barmherzigen Brüder Linz, Linz, Österreich; 7 ICMR – Institute for Cardiovascular and Metabolic Research, Johannes Kepler Universität Linz, Linz, Österreich; 8 1. Medizinische Abteilung mit Diabetologie, Endokrinologie und Nephrologie, Klinik Landstraße, Wien, Österreich

**Keywords:** Diabetes mellitus bei Erwachsenen, Lebensstil-Maßnahmen, Strukturierte Diabetesschulung, Patient:innen Empowerment, Patient:innen Selbtkontrolle, Diabetes in adults, Lifestyle measures, Structured diabetes education, Patient empowerment, Patient self-control

## Abstract

Diabetesschulung und Selbstmanagement nehmen eine zentrale Rolle in der Diabetesbetreuung ein. Das dabei angestrebte Patient:innen-Empowerment zielt auf die aktive Beeinflussung des Diabetesverlaufs durch Selbstkontrolle und Therapieanpassung sowie die Befähigung der Betroffenen, den Diabetes in ihren Alltag zu integrieren und an ihre Lebensumstände entsprechend anzupassen. Eine Diabetesschulung ist allen Personen mit Diabetes zugänglich zu machen. Um ein strukturiertes und validiertes Schulungsprogramm anbieten zu können, sind adäquate personelle, räumliche, organisatorische und finanzielle Voraussetzungen nötig. Neben dem Zuwachs an Wissen über die Erkrankung konnte gezeigt werden, dass eine strukturierte Diabetesschulung ergebnisorientiert Parameter wie Blutzucker, HbA_1c_, Blutfette, Blutdruck und Körpergewicht positiv beeinflussen kann. Neuere Schulungsmodelle betonen neben der Ernährung die körperliche Bewegung als wichtigen Bestandteil der Lebensstil-Therapie und bedienen sich interaktiver Methoden, um die persönliche Verantwortung herauszuarbeiten. Spezifische Situationen (z. B. verminderte Hypoglykämie-Erkennung, Krankheit, Reisen), das Auftreten diabetischer Folgeerkrankungen und der Einsatz technischer Geräte wie Glukosesensor-Systeme und Insulinpumpen bedürfen zusätzlicher Schulungsmaßnahmen unterstützt durch adäquate elektronische Hilfsmittel (Diabetes-Apps, Diabetes-Web-Portale). Neue Erkenntnisse belegen den Nutzen telemedizinischer oder internetbasierter Dienste für die Diabetesprävention und das Diabetesmanagement.

## Abstract (en)

Diabetes education and self-management play a critical role in diabetes care. Patient empowerment aims to actively influence the course of the disease by self-monitoring and subsequent treatment modification as well as the ability of patients to integrate diabetes into their daily life and to appropriately adapt diabetes to their life style situation. Diabetes education has to be made accessible for all persons with the disease. In order to be able to provide a structured and validated education program, adequate personnel as well as space, organizational and financial prerequisites are required. Besides an increase in knowledge about the disease it has been shown that a structured diabetes education is able to improve diabetes outcome as measured by parameters, such as blood glucose, HbA1c, lipids, blood pressure and body weight in follow-up evaluations. Modern education programs emphasize the ability of patients to integrate diabetes into everyday life, stress physical activity besides healthy eating as important components of life style therapy and use interactive methods in order to increase the acceptance of personal responsibility. Specific situations (e.g. impaired hypoglycemia awareness, illness, travel), the occurrence of diabetic complications and the use of technical devices such as glucose sensor systems and insulin pumps require additional educational measures supported by adequate electronic tools (diabetes apps and diabetes web portals). New data demonstrate the effect of telemedicine and internet-based services for diabetes prevention and management.

## Grundsatz-Statement

Der Diabetesverlauf hängt wesentlich vom Umgang der Betroffenen mit ihrer Erkrankung ab. Maßnahmen, welche sie befähigen, sich aktiv mit dem Diabetes auseinanderzusetzen, sind wichtiger Bestandteil jeder Behandlung. Angebot und Finanzierung der Schulung und Beratung sowohl im niedergelassenen Bereich als auch in den Krankenhäusern sollen in diesem Sinne sichergestellt sein.

## Zielsetzung

**Figure 1:**
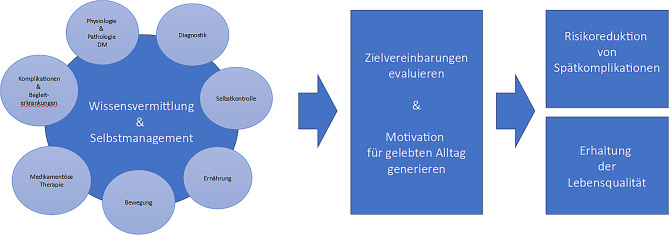

Die Diabetesschulung soll betroffene Personen zur Auseinandersetzung mit ihrer Erkrankung motivieren und ihnen Wissen, Fähigkeiten und Fertigkeiten vermitteln, welche für eine Umsetzung der Therapiemaßnahmen im Alltag zur Behandlung des Diabetes und möglicher Begleiterkrankungen und Komplikationen nötig sind, und damit die Erreichung individueller Behandlungsziele unterstützen. Betroffene werden über Diagnostik, Therapie (Ernährung, körperliche Aktivität, Medikation), mögliche Komplikationen, Begleiterkrankungen und Risiken bei Diabetes mellitus informiert. Diese Kenntnisse, Fähigkeiten und Fertigkeiten werden bei der Schulung im Zusammenhang mit dem Selbstbehandlungsverhalten überprüft (Abb. 1).

Moderne Ansätze in der Diabetesschulung bevorzugen dabei die Integration des Diabetes in den gelebten Alltag und bieten Bewältigungsstrategien zum Lösen persönlicher, sozialer und anderer Probleme an. Damit sollen sowohl die Lebensqualität erhalten als auch akute und chronische Folgen der Erkrankung verhindert werden. Langfristige Verhaltensänderungen im Sinne einer Lebensstilmodifikation sind damit ebenso gemeint wie das „Empowerment“ zum Selbstmanagement des Diabetes. Allgemeinmaßnahmen sind die Basis der Therapie und sollen bei Bedarf durch medikamentöse Interventionen begleitet werden. Die Definitionen von individuellen Zielen und Zielvereinbarungen sind wichtige Bestandteile im Behandlungsprozess. Jede Beratung im Sinne eines Coachings soll den unmittelbaren Vorteil – d. h. die unmittelbar erlebte Lebensqualität – einer guten Diabetestherapie täglich erkennen lassen, um langfristig ein komplikationsarmes Leben führen zu können [1–3].

## Indikationen

Es gibt fünf kritische Zeitpunkte, um die Notwendigkeit einer Schulung für Patient:innen mit Diabetes zu bewerten:

- Zeitpunkt der Diagnose
- Nicht Erreichen der Behandlungsziele im Rahmen der Kontrollen
- Umstellung von oraler auf parenterale Therapie
- Einsatz neuer Diabetestechnologie (Insulinpumpen, Glukosesensoren)
- Entwicklung von Komplikationen

Primär muss jeder/em Betroffenen baldmöglichst nach Diagnosestellung eines Diabetes die Teilnahme an einer strukturierten Diabetesschulung geboten werden. Bei Personen mit Typ-1-Diabetes geschieht dies üblicherweise im Rahmen des Beginns eine Insulinbehandlung stationär an einer geeigneten Krankenhausabteilung. Einige Wochen nach dieser „Grundschulung“ sollte die Möglichkeit gezielter individualisierter Information angeboten werden. Für Personen mit Typ-2-Diabetes steht die Schulung am Beginn einer Betreuung im Rahmen des Disease-Management-Programms „Therapie aktiv“, an welchem sich möglichst alle Ärzt:innen, welche Personen mit Typ-2-Diabetes betreuen, beteiligen sollten. Selbstverständlich wird eine Diabetesschulung aber auch Betroffenen angeboten, welche sich nicht in ein solches Programm einschreiben wollen. Auch spezialisierte Krankenhausabteilungen und Versicherer wie die ÖGK bieten dazu strukturierte Schulungskurse an. Bei Therapieänderung, insbesondere einer Umstellung von oraler Therapie auf eine parenterale Behandlung (wie GLP-1-Analoga und Insulin), soll unbedingt erneut eine Diabetesschulung erfolgen. Da Lebensstilmaßnahmen einer stetigen Auseinandersetzung damit bedürfen, sind wiederholte Schulungs- und Motivationsmaßnahmen notwendig und sinnvoll.

Inhalt und Umfang der Diabetesschulung:

- Hilfestellung zur Krankheitsakzeptanz
- Unterstützung zum eigenverantwortlichen Umgang mit der Erkrankung
- Beschreibung und Beurteilung von Therapiezielen
- Befähigung zur Integration der Diabetestherapie in den Alltag, insbesondere im sozialen Umfeld
- Kenntnisse über die Physiologie des Stoffwechsels
- Kenntnisse über die Grundlagen der Erkrankung (Ursachen, Symptome, Verlauf, Prognose), Beschreibung und Beurteilung von Therapiezielen

Interaktiv mit praktischen Beispielen unter Vermittlung von Information, Kenntnissen und praktischen Fertigkeiten:

- zu einer gesunden Ernährung in Abhängigkeit von BMI und Therapieform, unterstützt durch Einkaufstraining und -strategie
- zu körperlicher Aktivität und Sport
- zur Behandlung (Lebensstil- und medikamentöse Therapie)
- Erlernen von Selbstkontrolle und Anwendung notwendiger Maßnahmen (Blut- und Gewebszucker, Blutdruck)
- Erkennung und Behandlung von akuten Komplikationen (Hypoglykämie, Hyperglykämie, Infekte)
- Erkennung und Behandlung von diabetischen Folgeerkrankungen (Retinopathie, Nephropathie, Neuropathie, diabetischer Fuß)
- Erkennung und Behandlung von zusätzlichen kardiovaskulären Risikofaktoren (Blutdruck, Blutfette, Rauchen, Übergewicht) und Komplikationen (Herzinfarkt, Schlaganfall, periphere Durchblutungsstörung)
- zum Verhalten in besonderen Situationen (Krankheiten, Reisen, Festtage, körperliche Aktivität, Schwangerschaft, Stillzeit, Gestationsdiabetes, etc.)
- zu regelmäßigen Vorsorge- und Kontrolluntersuchungen (Gewicht, Blutdruck, Augen, Füße, Blutfette, HbA_1c_, Kreatinin, Mikroalbumin/Eiweiß im Harn, etc.)
- zu sozialrechtlichen Aspekten (Beruf, Versicherung, Führerschein, Status der Behinderung, Finanzausgleich etc.)

Zusätzlich bei Insulintherapie Vermittlung von:

- Kenntnissen, Fähigkeiten und Fertigkeiten zur Insulintherapie (Applikation, Dosisanpassung)
- Kenntnissen zu einer gesunden Ernährung und entsprechenden Interaktion zwischen Ernährung und Insulintherapie (Grundlagen Ernährung und Diabetes, Kohlenhydrat- bzw. BE-Berechnung, etc.) unterstützt durch Einkaufstraining und -strategie
- Kenntnissen zu körperlicher Aktivität, Sport und deren Auswirkungen auf die Erkrankung und die Insulintherapie
- Erkennung und Behandlung von akuten Komplikationen (Hypoglykämie, Hyperglykämie, Ketoazidose, Infekte)

Eine spezielle Schulung/Beratung für Personen mit:

- Gestationsdiabetes
- Insulinpumpen, Glukosesensoren
- Hypoglykämie-Erkennungsstörung etc.
- Anderen speziellen Diabetesformen (z. B. pankreopriver Diabetes, medikamentös induzierter Diabetes)

sollte in Diabeteszentren zusätzlich angeboten werden.

### Strukturelle Voraussetzungen

Um eine vergleichbare Schulungsqualität zu erreichen, sind neben inhaltlichen und methodischen auch räumliche, personelle und organisatorische Voraussetzungen nötig. Kriterien der Struktur‑, Prozess- und Ergebnisqualität sollten dazu erfüllt sein. Dies umfasst für die Struktur im niedergelassenen Bereich, Institut oder Krankenhaus die Beschreibung der Ziele, der Zielgruppe, der Art und Anzahl der Schulungseinheiten, der Teilnehmerzahl, der räumlichen Voraussetzungen, der Qualifikation der Schulenden, der Methodik und Didaktik, der Schulungsunterlagen und verwendeten Medien, der Maßnahmen zur Sicherung des Schulungserfolges und der Evaluierungsergebnisse. Die Prozessqualität muss durch ein Schulungsteam, üblicherweise bestehend aus Diabetesberater:in, Diätologe:in und Ärzt:in mit entsprechender Ausbildung gesichert werden. Die Ergebnisqualität der Schulungen sollte durch Kontrolle der Zielparameter Körpergewicht, Blutdruck, LDL-Cholesterin, Albumin/Kreatinin-Ratio im Harn, Blutzucker und HbA_1c_ festgestellt werden. Dazu ist die Beurteilung der Lebensqualität miteinzubeziehen. Für Patient:innen mit Typ-2-Diabetes erfolgt dies am besten im Rahmen der im DMP „Therapie aktiv“ vorgegebenen Quartalsuntersuchungen. Geschlechtsspezifische und ethnisch-kulturelle Aspekte sowie Sprachbarrieren sollten in der Schulung berücksichtigt werden, um eine nachhaltige Verbesserung der Stoffwechsellage zu erreichen [4, 5].

### Schulungsprogramme

Das Schulungs-Curriculum mit den oben genannten Inhalten kann dabei verschiedenen validierten Schulungsprogrammen folgen, welche an die vorherrschende Situation (Krankenhaus, Ordination, mobiles Schulungsteam) adaptiert werden können. Als Beispiel seien hier die auf dem „Düsseldorfer Schulungsmodell“ basierenden Programme „Behandlungs- und Schulungsprogramm für Typ 1 Diabetiker“, „Behandlungs- und Schulungsprogramm für Patienten, die nicht Insulin spritzen“ und „Behandlungs- und Schulungsprogramm für Typ 2 Diabetiker, die Insulin spritzen“ genannt, bei welchen die Wissensvermittlung im Vordergrund steht. Neuere Programme wie das an der MedUni Wien entwickelte DiabetesFIT Curriculum oder die in Bad Mergentheim/Deutschland entwickelten interaktiven Programme für Typ 1 „PRIMAS“ (Schulungs- und Behandlungsprogramm für ein selbstbestimmtes Leben mit Typ-1-Diabetes) und Typ 2 „MEDIAS 2“ (Mehr Diabetes Selbstmanagement für Typ 2) zielen auf das Empowerment der Patienten ab. Psychologische Aspekte des Alltagslebens untermauern diese Programme im Sinne einer Adaptierung des Diabetes an den individuellen Tagesablauf. Die Verwendung von multimedialen Schulungsmaterialien mit alltagsgerechten Beispielen unterstützt dies. Als stark interaktives Schulungsprogramm gelten die „Conversation Maps®“ („Gesprächslandkarten“) [6–8]. Diese sind ergebnisorientiert und flexibel auf Alltagssituationen aufgebaut. Diese Programme sind evidenzbasiert entwickelt und bauen auf internationalen klinischen Guidelines der IDF (International Diabetes Federation) auf [9]. Ein einfaches Programm zur Darstellung des Einflusses von körperlicher Bewegung auf den Blutzucker bietet z. B. DiSko („wie Diabetiker zum Sport kommen“), welches als zusätzliches Motivationsmodul in die Schulung eingebaut werden kann. Unterstützend können des Weiteren die Bewegungsbox und Ernährungsbox der ÖDG (www.ernaehrungsbox.at, www.bewegungsbox.at) eingesetzt werden. Psychologische Unterstützung sollte allen Patienten insbesondere bei Neumanifestation der Erkrankung und beim Auftreten von Komplikationen angeboten werden. Zusätzlich stehen für die Schulung von Komplikationen verschiedene Programme zur Verfügung: z. B. „HyPOS“ (Hypoglykämie – POsitives Selbstmanagement), Unterzuckerungen besser wahrnehmen, vermeiden und bewältigen. „Neuros“ (Aktiv werden – Neuropathie richtig behandeln), das neue Schulungs- und Behandlungsprogramm für Menschen mit Diabetes und Neuropathie. Programme zum Umgang mit technischen Neuerungen wie Glukosesensorsystemen und Insulinpumpen ergänzen diese (z. B. SPECTRUM für CGM, FLASH für FGM, INPUT für Insulinpumpentherapie) [10, 11].

Erkenntnisse der letzten Jahre belegen zusätzlich den Nutzen von Diabetes-Apps (z. B. MySugr) und tele-medizinischer oder internet-basierter Dienste (z. B. Diabetes Patientenfuchs) für die Diabetesprävention und das Diabetes-Selbstmanagement. Metaanalysen beschreiben dazu eine HbA_1c_-Verbesserung um 0,5 % [12–14, 27–33].

### Evidenzlage

Metaanalysen [15–18], ein NICE-Report [19] und ein bereits älterer Cochrane Review [20] können als Referenzen herangezogen werden. Letzterer gibt signifikante Effekte einer strukturierten Diabetesschulung an: HbA_1c_-Absenkung nach 12 Monaten um 0,8 %, Gewichtsreduktion um 1,6 kg, Reduktion des systolischen Blutdrucks um 2,6 mmHg, deutlicher messbarer Wissenszuwachs, jeweils im Vergleich zum Kontrollkollektiv. Vergleichbare Ergebnisse zeigen die Metaanalysen. Programme, welche das Selbstmanagement gegenüber einer reinen Wissensvermittlung betonen, schneiden dabei besser ab [1–3, 20–23]. Dies gilt ebenso für solche mit individualisierter Betreuung, Einbeziehung psychosozialer Komponenten, alters-angepasste Programme sowie die längerfristige Betreuung in Form von Einzelberatungen oder Nachschulungen in Gruppen [1, 6, 19–22]. Ein Standard liegt dazu beispielsweise von der Deutschen Diabetes Gesellschaft und der American Diabetes Association vor [1, 2]. Im Licht der zunehmenden Diabetesprävalenz werden Betreuungsprogramme mit wiederholtem Schulungsbedarf durch medizinisches Personal alleine kaum mehr zu bewältigen sein [24]. Disease-Management-Programme sind in der Diabetesbetreuung effektiv [25]. Peer-Support-Programme, in welchen Patienten selbst initiativ werden, um das Empowerment zu fördern, können unterstützend eingesetzt werden, zeigen aber uneinheitliche Effekte [26]. Selbsthilfevereine, zuletzt verbunden im Dachverein „Wir sind Diabetes“ bieten Betroffenen Unterstützung sowohl für die Bewältigung des Alltags als auch zu spezifischen Themen wie juristische Beratung oder Fragen zum Führerschein. In der Organisation von Gruppen-Treffen bieten sie verschiedene Möglichkeiten zum Austausch von Erfahrungen (Kinder- und Jugend-Camps, Lebensmittelkauf, Kochkurs, Insulin-Pumpen-Treffen, etc.) an.

## Interessenkonflikt

R. Weitgasser hat von folgenden Unternehmen, die teils auch fördernde Mitglieder der ÖDG sind, Forschungsunterstützungen und/oder Honorare erhalten: Abbott, Amgen, AstraZeneca, Boehringer-Ingelheim, Daiichi-Sankyo, Eli Lilly, Merck Sharp & Dohme, Novo Nordisk, Roche, Sanofi. C. Ciardi hat von folgenden Unternehmen, die teils auch fördernde Mitglieder der ÖDG sind, Forschungsunterstützungen und/oder Honorare erhalten: Abbott, AstraZeneca, Boehringer-Ingelheim, Eli Lilly, Sanofi-Aventis, Merck Sharp & Dohme, Novo Nordisk, Novartis. J. Traub hat von folgenden Unternehmen, die teils auch fördernde Mitglieder der ÖDG sind, Forschungsunterstützungen und/oder Honorare erhalten: Fresenius Kabi, Baxter Healthcare, Takeda Pharmaceutical, Nestle Österreich, Aengus Ernährungskonzepte. M. Riedl hat von folgenden Unternehmen, die auch fördernde Mitglieder der ÖDG sind, Forschungsunterstützungen und/oder Honorare erhalten: Sanofi-Aventis, Novo Nordisk, Boehringer Ingelheim, Eli Lilly, AstraZeneca, Medtronic. M. Clodi hat von folgenden Unternehmen, die auch fördernde Mitglieder der ÖDG sind, Forschungsunterstützungen und/oder Honorare erhalten: Abbott, AstraZeneca, Bristol-Myers Squibb, GlaxoSmithKline Pharma, Eli Lilly, Merck Serono, Merck Sharp & Dohme, Novartis, Novo Nordisk, Roche, Roche diagnostics, Sanofi-Aventis, Servier, Takeda. B. Ludvik hat von folgenden Unternehmen, die auch fördernde Mitglieder der ÖDG sind, Forschungsunterstützungen und/oder Honorare erhalten: Abbott, Amgen, AstraZeneca, Bayer Health Care, Boehringer-Ingelheim, Bristol-Myers Squibb, Germania Pharmazeutika, GlaxoSmithKline Pharma, Johnson & Johnson Medical, Eli Lilly, Medtronic, Merck Serono, Merck Sharp & Dohme, Novartis, Novo Nordisk, Pfizer, Roche, Sanofi-Aventis, Servier, Takeda, Metacure. M. Barta gibt an, dass kein Interessenkonflikt besteht.
